# Prolonged and Tedious Labors and Forceps Deliveries Compared as Causes of Epilepsy, Idiocy and Cerebral Diplegias1Read before the Buffalo Academy of Medicine, December 8, 1908.

**Published:** 1909-02

**Authors:** James Wright Putnam

**Affiliations:** Buffalo. N. Y.; Professor of nervous diseases, University of Buffalo, Ex-president American Neurological Association


### BUFFALO MEDICAL JOURNAL.

VOL. LXIV. FEBRUARY, 1909. No. 7

### ORIGINAL COMMUNICATIONS.

### Prolonged and Tedious Labors and Forceps Deliveries Compared as Causes of Epilepsy, Idiocy and Cerebral Diplegias.[1]

BY JAMES WRIGHT PUTNAM, M.D., Buffalo, N. Y,

Professor of nervous diseases, University of Buffalo, Ex-president American Neurological Association.

A STUDY of this question has seemed to me of sufficient importance to make a search of the literature for the purpose of finding the views of various writers in different countries as to the relative proportion of cases caused by these factors.

Epilepsy is so dreadful an affliction and, withal, is so prevalent that any means of lessening the number of its victims is a decided gain for the community. It is estimated that there is one epileptic for every 1,000 of the population and that would mean 400 in Buffalo.

Idiocy, that state of mental deficiency dating from birth and lasting through life, is an equally great affliction. The census of 1900 places the number of idiots in the United States as 95,000.

Infantile cerebral palsies are those paralytic affections which date from birth. They were originally described by Little of England, and are known generally as Little's Disease. These three conditions may all be the result of meningeal hemorrhage occurring at the time of birth. It is necessary therefore to enquire into the causes of meningeal hemorrhage in infants newly born. Little reported sixty-three cases in all of these abnormal conditions attended the birth. One was born cyanosed; in one, turning was performed; another had the cord turned about the neck; another was a foot presentation and was unconscious for two hours after birth; in all of these there was evidence of pressure and paralysis. In 1882, twenty years later Ross, of England, reported several cases in which the difficulty resulted from foot presentation. In one case there was foot presentation, the labor

1. Read before the Buffalo Academy of Medicine, December 8, 1908.

was difficult and convulsions with the paralysis immediately followed the delivery.

To an American, Dr. Sarah McNutt, belongs the honor of first proving this paralysis was due to meningeal hemorrhage. Her article was published in the *American Journal of Medical Sciences,* 1885, and has since been quoted by all writers on this subject. Aside from foot presentations, prolonged and tedious labors without the aid of forceps is ascribed as a cause; also labors which were precipitate and labors in which the forceps were used. Asphyxia is assigned as the only cause in many cases. That we may form an opinion as to the relative importance of these, I read the following quotations taken from obstetricians, pediatrists and neurologists.

OBSTETRICIANS.

Dr. Kauffman consulted over forty works on obstetrics for me with reference to quoting them on the question of the comparative harm to the child from the use of forceps, as compared with prolonged difficult labors. The result of his search was that only one author expressed any opinion on the subject or considered it in any way.

They agree, however, in the four indications for the use of the forceps from the standpoint of the child: (1) forceps should be applied if the pulse is under 100 or over 160; (2) if there are sudden marked violent movements of the child; (3) if meconium appears during head presentation; (4) if there is irreplacable prolapse of the funis. These indications are for the preservation of the life of the child. Indications for the application of forceps with reference to the future mental and physical condition of the child is mentioned by E. P. Davis, of Philadelphia in his treatise on obstetrics, p. 740 to 742:

It is noticeable that most of the cases of congenital epilepsy and idiocy develop in children who were not delivered by forceps, but in whom birth was greatly prolonged, although spontaneous. This illustrates the value of forceps as a conservative instrument and emphasises the fact of the necessity for their proper employment. It also draws attention to the value of other obstetric operations which deliver the mother without the difficult and prolonged passage of the child through the unaltered pelvis.

How this opinion of an obstetrician is held in harmony with clinicians in other specialties these quotations will show. Dr. Van Peyma in a valuable paper read before this academy in 1907, said: It should be known that mutilated, mentally enfeebled, paralytic, and dead children; lacerated, invalided and dead mothers are the ever recurring product of unintelligent and conscienceless obstetric practice.

PEDIATRISTS.

Rotch, of Boston, Professor of Pediatrics in Harvard Medical School, in his classic work on pediatrics, says:

Idiocy is due to pressure on the head at birth; and that cerebral palsy results from asphyxia, in tedious prolonged deliveries rather from forceps. (Ed. 1901.)

Holt, 3 edition, 1906, p. 108, chapter on birth paralysis:

Birth paralyses are chiefly due to pressure on the child by the parts of the mother, or by artificial means of delivery. The conditions with which they are associated are tedious labor, breech presentations with difficulty in extracting the head, instrumental deliveries, and premature births.

Taylor and Wells, Diseases of Children, 1901, p. 539, under Infant Cerebral Palsy, say:

The chief cause of paralysis during parturition is a slow labor, exercising a long continued compression on the fetal brain and circulation, producing meningeal hemorrhage or thrombosis. This is most common in first children. The use of instruments to relieve this tardiness, though it may occasionally work havoc, yet affords rather a means of prevention in most instances by relieving the prolonged intracranial blood pressure.

A. Jacobi, the Dean of American Pediatrists in the *Archives of Psychiatry,* 1890, in an article calls attention to the fact that many children who are born asphyxiated are physically and mentally incapable of healthy and normal development owing to faults of structure. Not infrequently, such children who are resuscitated after long continued effort live only a few hours or days, or, if they survive, develop into idiocy. While it is admitted that the improper or unskilful use of forceps produces results of this character in some instances, he believes they are oftener due to delay in the use of forceps.

Oppenheim, Diseases of the Nervous System, p. 533, says:

Forceps delivery is also blamed, although it does not appear to be as much the use of the instrument as the factor which renders it necessary.

Church-Peterson, 6 edition, 1908, p. 243:

The conditions attending birth frequently lead to brain lesions in the child. The great majority of cerebral birth palsies occur in protracted labors and consequently in primiparæ. A number of them occur in precipitate labor and in both are due to violent compression of the fetal head. Comparatively few cases can be attributed to the use of forceps, and it is exactly this class of cases that the labor is apt to have been tedious. Forceps accidents, however, cannot be denied or overlooked, and the misuse of these powerful instruments is fraught with serious results to the skull and brain of the child. The facts, however, favor early skilful instrumental interference as compared with tedious labor.

The same authors in the chapter on Idiocy, page 876, say:

Parturitional factors (meningeal hemorrhage, from prolonged labor, asphyxia at birth, premature birth, pressure on the cord, forceps injuries, etc.) are active in about 18 per cent. It may be said that forceps are far less dangerous to the child than tedious labor.

Mills, in his work, 1898, chapter on Cerebral Paralyses of Childhood, p. 615, says:

Prolonged and difficult labor, particularly with first born children is one of the most clearly demonstrated causes of cerebral palsies. While the direct application of force by instruments may inflict injuries on the head and brain, these cerebral affections are far more frequently due to compression of the child's head in the pelvic strait, and to the asphyxia which results from protracted labor. Skilfully applied forceps, indeed, in some cases prevents the occurrence of these unfortunate palsies.

Collins, Nervous Diseases, 1904, p. 181, says:

It is unnecessary to enumerate all the causes which contribute to the encephalopathies of natality. They may be summarised in one word—dystocia. The liability of the brain of the child at the time of birth is in direct proportion to the duration of the labor. The dangers of dystocia are usually considered relative to the life of the child, but the urgency is very great that they be considered relative to the occurrence of infant cerebral palsy. The injuriousness of the forceps delivery is slight when compared with the far reaching effects of tedious labor.

Dana, Chapter LI, p. 489. Those cases of cerebral palsy that occur at the time of birth are due to tedious labor, the use of forceps, and other injuries at the time of labor.

Starr, 2 edition, p. 521, chapter on Cerebral Atrophies of Childhood, says:

Many cases date from birth. Thus in 177 of my cases (400), there was a history of difficult labor, or a malposition requiring manual delivery, or instrumental delivery. In several cases labor was premature, and the child was kept alive with difficulty. In two cases the patient was a twin. In many cases the child was born asphyxiated. In all these cases it is probable that meningeal or cerebral hemorrhage occurred during delivery, and the pressure upon the brain prevented normal development.

Starr, p. 742, Epilepsy: Injuries of the brain, causing hemorrhages either gross or capillary, followed by the development of cicatricial tissue are a very common cause of epilepsy. That such injuries occur in the process of labor by undue compression of the head or by a delay in the delivery, the formation of a large caput and a corresponding congestion of the brain is admitted. This is the most common cause of the cerebral atrophies of childhood in which epilepsy develops in a large per centage of cases. No surprise is expressed when epilepsy appears in these cases where a gross lesion is apparent. But many cases of epilepsy date from birth. Here the hemorrhage is so small as to leave no evidence of paralysis or of sensory defect, or of mental deficiency, but has been sufficient to form a focus of irritation in the brain.

Dercum, p. 507: Cases having their origin in trauma during birth is quite large. The head of the child in its passage through the pelvis is exposed to varying degrees of pressure, and it cannot cause surprise if some times, for example, when this pressure has been excessive or unduly prolonged, that cranial lesions ensue. It is not impossible that the prolonged asphyxia during birth is a factor in the subsequent cortical degeneration. A few cases of cerebral palsy have been traced to injury by the forceps. Osler had nine cases of such injury due to this cause in one hundred and twenty infantile hemiplegias. It is certain, however, that the injury is due less frequently to the forceps than to the dystocia which renders their employment necessary.

Sachs, p. 436: The etiology of birth palsy is a very simple one. It is surprising how much pressure the brain and skull will tolerate without injury, but it is natural that harm should occasionally be done. My own studies, based on the study of a very large number of statistics, have proved that tedious labor is a more frequent and more disastrous factor than instrumental delivery. They occur most frequently in first born children. If physicians were more confident in their ability to apply forceps, protracted labor would not be as powerful an etiological factor as it is at the present time. There is, therefore, a distinct inference to be drawn from these facts, and a word of warning should be uttered to the obstetrician that, other things being equal, and, above all, the life of the mother not being in danger, it is wise to curtail the period of labor as much as possible, and not necessarily to wait until the child's heart's action becomes feeble. Many children might have escaped idiocy and epilepsy if the period of labor had been properly managed.

Pierce-Bailey in American System of Practical Medicine, Loomis and Thompson, 1898, Vol. 4, p. 861:

The factors which are active at the time of birth in the causation of idiocy are mainly of a traumatic character, and are such as may bring about direct injury of the infant brain or its membranes, or such as may obstruct the circulation for too long a time. Thus prolonged parturition by causing protracted pressure on the ununited bones of the skull may seriously compress the brain or may induce asphyxia. Compression of the head by forceps figures in a certain proportion of cases.

Osler, in Pepper's System, Vol. I., speaking of cerebral palsies, page 711, says:

The possibility of injury to the brain must be borne in mind in case of protracted labor. Probably a long continued compression of the head entails greater risks than the application of the forceps.

Berkley, Johns Hopkins, in his treatise on Mental Diseases, page 492, says:

Difficult delivery, with compression and injury of the infants skull at birth, is the cause of a certain proportion of cases of idiocy. The use of the forceps in delivery apparently plays a very unimportant part in the cephalic injuries, long continued compression being far more injurious and deleterious to the child than properly conducted artificial extraction.

Wulff in 1,436 cases of idiocy found 11.9 to be due to injury to the skull at or about the time of birth.

Christian A. Herter in Diseases of Nervous System, page 389, cerebral birth palsy, says:

This form of infantile cerebral birth palsy depends on meningeal hemorrhage over one or both sides of the convexity of the brain or on its base, the results of injury during birth. There is a history of some distinct difficulty in birth, either an unnatural presentation or more frequently prolonged and difficult labor with head presentation in primipara. Comparatively few instances can be attributed to forceps delivery alone. Precipitate delivery produces compression of the head similar to that occuring during prolonged labor.

Stewart Paton of Johns Hopkins in his book on Psychiatry, 1905, page 202 quotes: Fletcher Beach, "in examining 810 idiots, found that 35 cases showed that the injury was due to the use of forceps, and that 216 cases were cases of difficult labor without instrumental delivery."

Kuntzel, in 500 cases of idiocy, estimated 8.9 per cent. forceps had been used; a difficult but noninstrumental delivery was a factor in 4.5 per cent.

Bianchi of Naples, in his book, page 463, says:

Injuries of the head during pregnancy and difficult parturition, with prolonged compression of the head and consecutive lesions (meningeal or even cerebral hemorrhage) and injuries to the head after birth, are perhaps among the most frequent causes of the arrest of development of the brain.

From Transactions of the Obstetrical Society of London, Vol. 18, p. 298-300 (1876). I quote:

THE OBSTETRICAL ASPECTS OF IDIOCY, BY J. L. DOWN, M. D.

I think it can not fail to strike the fellows of the society as a very important fact that no fewer than 20 per cent. of the idiots of my investigation were born with well-marked symptoms of suspended animation—symptoms which required marked and strenuous efforts for their removal and to effect resuscitation. Moreover, it is specially interesting that while, as we have said, 24 per cent. of idiots generally are primiparous, as many as 40 per cent. of resuscitated idiots come under this category. Clearly, then, suspended animation is a condition as far as possible to be avoided by the practical obstetrician. Bearing on this part of the subject is the question of instrumental interference. I have made a careful inquiry on this subject over a large area, and with the expectation I should find a large amount of idiocy to be the result of "meddlesome midwifery," but I am bound to say that my statistics falsify my anticipations, and redound to the care with which instruments are employed in parturition. I find from an examination of all my cases about which positive information could be obtained that in only 3 per cent. were the forceps employed. In every case, however, where they were employed the friends of the child believed that the instrument alone was the cause of the disaster, while in nearly every case I could discover quite sufficient to account for the mental defect in the neurotic history of the progenitors. In a very few cases, only a small fractional percentage could I arrive at the conclusion that the use of forceps was the principal cause of the calamity.

I find in a very large number of my cases that the labor was stated to be unusually tedious and prolonged. Dr. Playfair has shown that the employment of the forceps does not interfere with the viability of the offspring, and that a prolonged labor is more compromising to the life prospects of the child than a judicious and timely application of the forceps.

Certainly my statistics make clear to me, contrary to what some years since was advanced by Dr. Arthur Mitchell and in harmony with Dr. Ramsbotham's experience, that the use of the forceps is not an important factor in the production of brain disease. When, however, it is borne in mind how frequent is the association of suspended animation with idiocy, it cannot, I think, be too strongly enforced that the mental integrity of the child is more likely to be compromised by a prolonged pressure in the maternal passages than by skilled employment of artificial assistance. The accoucheur who postpones instrumental help often does so at the risk of terrible consequences to the nervous system of the little one he is solicitous to protect.

G. E. Shuttleworth in Vol. VIII, page 32, of Encyclopedia Medica in the article on mental deficiency says:

Birth palsies due to intracranial pressure at the time of birth. While instrumental delivery has been blamed by certain writers, it is probable that protracted pressure in an unassisted, unduly prolonged labor is a much more potent factor in causation of meningeal hemorrhage and mental defect; in fact in my statistics and those of Dr. Beach, protracted labor figures more than four times as often as forceps deliveries in causation of mental defect.

In the Royal Albert Asylum for Idiots, England, the assigned cause of idiocy in 29 per cent. of the admitted cases is prolonged labor without instrumental interference. *British Medical Journal,* January 30, 1886.

Crichton Browne in the West Riding Asylum, advances the opinion that protracted and abnormal labors are an important factor in the causation of idiocy.

Mitchell, quoted by Sachs, in his chapter on idiocy, page 544, reported 57 cases of idiocy in which labor lasted more than thirty-six hours, 22 cases in which forceps were applied, 9 of which showed the impression of the forceps on the head after birth; 4 were born by version; 6 were breech presentations; 9 were born prematurely; 29 were born asphyxiated and supposed to be dead. In this record, we see that tedious labors were 57 forceps cases, 22 less than one-half.

Fisher, Edward D., in the same work, in the article on cerebral palsies of infancy, p. 400, says:

A very common cause and one of much importance to the general practitioner is of traumatic origin, due to pressure of the maternal parts in tedious delayed labors, or from an abnormally narrow pelvis, or an unusually large head on the part of the child, or again in the use of the forceps. The last cause is less frequent than is generally supposed. Delayed labor, even without abnormally of mother or child is more frequently the cause. It is wiser, therefore to apply the forceps early, rather than to submit to the dangers of compression.

Gowers, page 415, in his diseases of the Nervous System, says:

The hemorrhage that injures the brain is almost always due to special difficulty in parturition. In about one fifth, the head is born last, and in such cases the intense mechanical compression makes the occurrence easy to understand. The same symptoms occur in cases of head presentation when the labor has been long and difficult, and the immediate effects were shown by the difficulty in getting the child to live, or by distinct symptoms of convulsions, rigidity, immobility of some part or dysphagia, present from birth or coming on a few days after. About one-half the cases were first born but in almost all the others the labor was specially difficult. In some of the cases the forceps were used, but it is probable that as a rule the lesion was the result not of the use of the instruments but of the conditions that made them necessary. In rare cases the clumsy use of the instruments has produced the injury, as in one instance in which a permanent indentation corresponded to the center of the opposite arm which was affected. But it is most important that the fact should be clearly recognised, that the result is not due to the instrumental aid.

This research shows that the obstetrician has a much more important function than merely bringing a live child into the world. We also demand of him that he so perfect himself in the science of obstetrics that the preventable deformities, idiocies, and epilepsies shall be reduced. When we realise that there are in the United States upward of 95,000 idiots and more than 123,000 epileptics, that many of these are so afflicted from the preventable accidents of birth, these quotations taken from the writings of eminent men in Europe and the United States, show with marvelous unanimity. There is not a dissenting voice. All agree that, speaking generally, idiocy, epilepsy and cerebral palsies of childhood are due to one of three causes, asphyxia, injuries to the head from forceps, and injuries from prolonged compression. Let us hope that this truth will so impress itself on the profession that these accidents of birth may be reduced to a minimum.

525 Delaware Avenue.

